# Quality of life of patients with dermatologic disease in Bagamoyo, Tanzania: A cross-sectional study

**DOI:** 10.1016/j.jdin.2026.09.013

**Published:** 2026-10-05

**Authors:** Natalie B. Norton, May Madi Han, Lisa Rein, Kai Yang, Connor R. Linzer, Mariah C. Estill, Magreth Lukwaro, Magdalena Dennis, Gasper Mmbaga, Jane J. Mcharo, Stephen R. Humphrey, Omar Juma, Karolyn A. Wanat

**Affiliations:** a Medical College of Wisconsin, School of Medicine, Milwaukee, Wisconsin; b Division of Biostatistics, Medical College of Wisconsin, Data Science Institute, Milwaukee, Wisconsin; c Department of Dermatology, Medical College of Wisconsin, Milwaukee, Wisconsin; d Bagamoyo District Hospital, Bagamoyo, Tanzania; e Muhimbili National Hospital, Dar es Salaam, Tanzania; f Ifakara Health Institute, Bagamoyo, Tanzania

**Keywords:** cross-sectional study, DLQI, global health, international dermatology, patient-reported outcomes, quality of life, resource-limited, SDOH, skin disease, social determinants of health, Tanzania

Dermatologic diseases disproportionately impact quality of life (QoL) in resource-limited settings; however, this effect remains understudied in Sub-Saharan Africa.^1^ The high prevalence of skin disease and rates of underdiagnosis in Tanzania further increase the potential for chronic disease progression and disease burden on QoL.^1^, ^2^ This study aimed to identify which diseases, characteristics, and demographic factors were associated with the greatest impact on QoL.

This cross-sectional study was conducted on a convenience sample of patients in Bagamoyo, Tanzania from November 2024 to July 2025. A total of 174 of 181 approached patients (96% response rate) reviewed an informational letter and provided informed consent before completing the Dermatology Quality of Life Index (DLQI) and a demographics survey. The primary analysis modeled DLQI score as an ordinal outcome using proportional odds logistic regression. Study variables were also examined by DLQI categories: 0 to 5 (no to small effect) versus ≥6 (moderate to extremely large effect), using the Wilcoxon rank-sum test for continuous and ordinal variables and Fisher’s exact test for categorical variables. The dichotomized DLQI outcome was analyzed using logistic regression as a sensitivity analysis. Variables selected a priori based on clinical expertise and the literature were included in multivariable adjusted models. Analyses used an available-case approach for missing data (2.5%), with the number of observations reported for each statistical test.

Analysis included 163 participants with complete DLQI scores who were 69% female with a median age of 35.5 years (range, 16-90 years) (Table I). The mean DLQI score was 7.52 ± 6.99, with 48% of participants reporting a score of ≥6. The most prevalent patient-reported factors were itch (56%), pigmentation changes (16%), presence of acne/rash (14%), and facial involvement (7%). The most common diagnoses were fungal infections (30%), bacterial infections (20%), acne (18%), and eczema (14%).

**Table I:** Patient demographics based on Dermatology Quality of Life Index score

Characteristic	Overall	DLQI 0-5	DLQI ≥6	*P* value
	*n* = 163∗	*n* = 85†	*n* = 78†	
The age category, y				.272‡
16-25	42 (26)	19 (45)	23 (55)	
26-39	53 (33)	29 (55)	24 (45)	
40-59	58 (36)	29 (50)	29 (50)	
60-79	8 (4.9)	7 (88)	1 (13)	
80-89	1 (0.6)	1 (100)	0 (0)	
90+	1 (0.6)	0 (0)	1 (100)	
Biological sex				.866§
Male	51 (31)	26 (51)	25 (49)	
Female	111 (69)	59 (53)	52 (47)	
Missing	1	0	1	
Marital status				.329§
Married	90 (56)	41 (46)	49 (54)	
Widowed	7 (4.3)	5 (71)	2 (29)	
Divorced	3 (1.9)	2 (67)	1 (33)	
Separated	10 (6.2)	6 (60)	4 (40)	
Never married	51 (32)	31 (61)	20 (39)	
Missing	2	0	2	
Highest level of education				.346‡
No education	6 (3.7)	2 (33)	4 (67)	
Primary	76 (47)	39 (51)	37 (49)	
Secondary	50 (31)	27 (54)	23 (46)	
Associate	15 (9.3)	9 (60)	6 (40)	
Bachelor	13 (8.1)	7 (54)	6 (46)	
Master	0 (0)	0 (0)	0 (0)	
Doctorate	1 (0.6)	1 (100)	0 (0)	
Missing	2	0	2	

Patient demographics are represented by *n* (% of overall total) and *n* (% of row total) based on DLQI scores. A Wilcoxon rank-sum test was performed for each continuous or ordinal variable. A Fisher’s exact test was performed for each categorical variable.

*DLQI*, Dermatology Quality of Life Index.

∗ *n* (% of overall).

† *n* (% of row total).

‡ Wilcoxon rank-sum test.

§ Fisher’s exact test.

In the adjusted ordinal model, location, increased travel as a barrier to care, hypopigmentation, and level of concern regarding treatment affordability were significantly associated with higher DLQI scores independent of other variables (*P* < .05) (Table II). Additional analysis using a DLQI cutoff of ≥6 as a point of clinical significance (moderate to severe impact on QoL), location, increased travel as a barrier to care, eczema, hypopigmentation, and distribution on the legs were significantly associated with impact on QoL (Supplementary Table I, available via Mendeley at https://data.mendeley.com/datasets/3bgd5z25yh/2).

**Table II:** Adjusted ordinal logistic regression for total Dermatology Quality of Life Index score

Characteristic	aOR	95% CI	*P* value
Location			
Bagamoyo	-	-	
Other	4.09	2.19, 7.78	**<.001**
Diagnosis: eczema	1.84	0.80, 4.33	.154
Symptom: hypopigmentation	3.67	1.25, 11.0	**.019**
Body part: legs	1.81	0.93, 3.59	.084
Symptom: itch	0.80	0.44, 1.45	.460
Less travel would make it easier to receive dermatologic treatment	2.80	1.49, 5.31	**.001**
How concerned do you feel about being able to afford treatment for your skin condition(s)?			
None to slightly concerned	-	-	
Moderately concerned	2.52	1.36, 4.70	**.003**
Extremely concerned	9.23	3.36, 26.7	**<.001**

For categorical predictor variables, an odds ratio > 1 indicates higher odds of having a larger DLQI score (worse effect on quality of life) compared with the reference category after adjusting for all other variables in this table. Bolded values indicate statistical significance (*P* < .05).

*aOR*, Adjusted odds ratio; *DLQI*, Dermatology Quality of Life Index.

Previous studies have found significant associations between QoL and atopic dermatitis, pigmentation changes, and social determinants of health (SDOH), which are consistent with our findings.^3^, ^4^ For the 40% who reported that reduced travel would improve access to care, 69% had a DLQI ≥6. One potential explanation for this association is that limited access to transportation may prevent patients from seeking out early and preventative care, which has been associated with more severe presentations of skin diseases that may serve as initial indicators of systemic illness.^1^, ^2^

High levels of heat and humidity in tropical climates may exacerbate the severity of symptoms associated with eczema, which commonly involves the legs, and can result in persistent postinflammatory hypopigmentation that is often more pronounced in darker skin tones.^5^ Impact on clothing choice, reported by 46.8% of participants, has been significantly associated with decreased QoL.^1^, ^4^ Clothing practices and materials worn may influence symptom burden through skin occlusion and reduce social impact on QoL through disease concealment.

These results reinforce the importance of future collaborative projects addressing SDOH, which may serve as important correlates in the relationship between dermatologic disease and QoL. Limitations include a single district in Tanzania (Bagamoyo), inability to determine causality and chronicity, reliance on patient self-reporting, and narrow disease representation. Further research is needed to explore additional SDOH and cultural factors that may influence dermatology-related QoL in this population.

### Declaration of generative AI and AI-assisted technologies in the writing process

None.

## Conflicts of interest

None disclosed.
